# Traumatic Injury among Patients Presenting to the Department of Emergency Medicine of a Tertiary Care Centre

**DOI:** 10.31729/jnma.8423

**Published:** 2024-02-29

**Authors:** Suraj Rijal, Darlene Rose House, Nishant Joshi, Barsha Thapa, Kriti Shrestha, Mohan Raj Sharma

**Affiliations:** 1 Department of General Practice and Emergency Medicine, Patan Academy of Health Sciences, Lagankhel, Lalitpur, Nepal; 2 Departments of Global Health and Emergency Medicine, Icahn School of Medicine Mount Sinai, Gustave L. Levy Pl, New York, United States of America; 3 Department of Neurosurgery, Institute of Medicine, Tribhuvan University Teaching Hospital, Maharajgunj, Kathmandu, Nepal

**Keywords:** *emergency care*, *injuries and wounds*, *injury severity score*, *trauma unit*

## Abstract

**Introduction:**

The majority of trauma-related deaths occur in low- and middle-income countries; however, limited data exists in these settings related to injury types and severity. The prevalence of trauma similar to our setting was less estimated. This study aimed to find the prevalence of traumatic injury among patients presented to the department of emergency medicine of a tertiary care centre.

**Methods:**

This is a descriptive cross-sectional study conducted among patients presented to the Department of Emergency Medicine from 15 September 2021 to 14 September 2022. Ethical approval was taken from the Institutional Review Committee. World Health Organization trauma minimum data set, injury mechanism, types and patient disposition data were collected and injury severity scores were calculated. A convenience sampling method was used. The point estimate was calculated at a 95% Confidence Interval.

**Results:**

Among 47,825 patients, 1,524 (3.19%) (3.03-3.34, 95% Confidence Interval) patients presented with a traumatic injury. A total of 967 (63.45%) were males and had a median age of 30 years (Interquartile range: 25). Most injuries were caused by falls 650 (42.65%), followed by road traffic accidents 411 (26.97%). A majority had minor Injury Severity Scores 1280 (83.99%).

**Conclusions:**

The prevalence of traumatic injury among patients presenting to emergency was found to be lower than other studies done in similar settings.

## INTRODUCTION

Annually, over five million deaths occur due to trauma-related injuries, with 90% of these fatalities occurring in low and middle-income countries (LMICs).^1^ As a lower-middle-income country, Nepal faces growing vulnerability to injuries due to frequent natural disasters and rapid industrialization. Recent data reveals a rising public health burden, evident in increasing injury-related deaths and disability-adjusted life years (DALYs).^2^

There have been several hospital-based studies exploring injuries in Nepal; however, these are primarily convenience samples and are likely to underestimate the true injury burden.^3^ Furthermore, most of these published studies provide less information regarding injury types and severity.

With traumatic injuries on the rise, there is a call for more accurate reporting and documentation. Improved accuracy in reporting will aid in developing a clearer picture of the prevalence trends over time.

This study aimed to find the prevalence of traumatic injury among patients presented to the department of emergency medicine of a tertiary care centre.

## METHODS

A descriptive cross-sectional study was conducted among patients presented to the Department of Emergency Medicine of Patan Academy of Health Sciences, Lagankhel, Lalitpur, Nepal. Data of patients from 15 September 2021 to 14 September 2022 were collected after obtaining ethical approval from the Institutional Review Committee of the same institute (Reference number: drs2106111534). Patients presenting to the Department of Emergency Medicine during from 15 September 2021 to 14 September 2022 were included in the study. Incomplete data were excluded from the study. A convenience sampling method was used. The sample size was calculated by using the following formula:


$$\begin{array}{ll} \text{n} & ={\text{Z}}^{2}\times \frac{\text{p}\times (1-\text{p})}{{\text{e}}^{\text{2}}}\\ & =1.96^{2}\times \frac{0.50\times (1-0.50)}{0.05^{2}}\\ & =9605 \end{array}$$

Where,

n = minimum required sample size
Z = 1.96 at 95% Confidence Interval
p = prevalence taken as 50% for maximum sample size calculation
q = 1-p
e = margin of error, 1%

The calculated minimum required sample size was 9605. As the convenience sampling method was employed the sample size was quadrupled and the sample size calculated was 38,420. However, a total of 47,825 patients were included in this study.

Data was collected based on the WHO MDI and Injury severity score (ISS).^3-4^

Data were entered and analysed using Microsoft Excel 2019. The point estimate was calculated at a 95% CI.

## RESULTS

Among 47,825 patients, 1524 (3.19%) (3.03-3.35, 95% CI) patients presented with a traumatic injury. A total of 967 (63.45%) were males and had a median age of 30 years (IQR 25) (Table 1).

**Table 1:** Demographics of trauma patients presented in ED (n = 1524).

Demographics	n (%)
**Age (years)**	
<18	285 (18.70)
18-29	457 (29.99)
30-39	249 (16.34)
40-49	210 (13.78)
50-59	120 (7.87)
60-69	90 (5.91)
>70	113 (7.41)
**Sex**	
Male	967 (63.45)
Female	557 (36.55)

Most injuries were caused by falls 650 (42.65%), followed byroad traffic accidents 411(26.96%) (Table 2). Injuries wereunintentional in 1403 patients (92.06%), intentional in 85(5.57%), and related to self-harm in 36 (2.36%).

**Table 2:** Mechanism of traumatic injury (n = 1524).

Mechanism of Injury	n (%)
Fall	650 (42.65)
Road traffic injury	411 (26.97)
Cut	227 (14.90)
Assault	98 (6.43)
Burn	23 (1.51)
Crush	23 (1.51)
Stabbing	6 (0.39)
Choking/Hanging	6 (0.39)
Drowning	2 (0.13)
Others^*^	78 (5.12)

* Others: Electrocution, dislocation, pulled elbow, tympanic membrane perforation and injuries due to self-manipulation via foreign bodies, penile fracture, dog bite, snake bite, rat bite, abrasions, ring avulsion injuries, soft tissue injuries due to various mechanisms.

Most injuries involved extremities including the pelvis 1,128 (74.02%), followed by external surfaces 571 (37.47%) and the head and neck 251 (16.47%) (Table 3).

**Table 3:** Injuries according to location and severity (n = 1524).

Injury Category	Head and Neck n(%)	Face n(%)	Chest n(%)	Abdomen n(%)	Extremity (Including Pelvis) n(%)	External n(%)
Minor	168 (11.02)	120 (7.87)	60 (3.94)	30 (1.97)	547 (35.89)	484 (31.76)
Moderate	20 (1.31)	335 (21.98)	32 (2.10)	10 (0.66)	434 (28.48)	81 (5.31)
Serious	17 (1.12)	1 (0.07)	6 (0.39)	19 (1.25)	138 (9.06)	4 (0.26)
Severe	29 (1.90)	2 (0.13)	-	2 (0.13)	4 (0.26)	-
Critical	13 (0.85)	1 (0.07)	1 (0.07)	2 (0.13)	5 (0.33)	1 (0.07)
Unsurvivable	4 (0.26)	1 (0.07)	1 (0.07)	-	-	1 (0.07)

Also, most patients had an ISS that was minor 1280 (83.99%). Regarding disposition, 1102 (72.31%) patients were discharged, 318 (20.87%) were admitted, 99 (6.50%) were referred, and 5 (0.32%) patients died in the emergency department.

## DISCUSSION

In this study, 1524 (3.19%) patients presented with a traumatic injury. In a similar study done in a nationwide population survey, the prevalence was 13.1%.^5^ Another similar study showed the prevalence of minor injury was 3.1% while 0.4% of major injuries 0.4 %. ^6^

The predominant mechanism of injury was related to falls followed by RTIs. Similar studies done in Nepal have also found falls from height as the most common mode of injury followed closely by RTIs.^7,8^ One epidemiological study from Nepal found higher rates of RTIs followed by falls.^9^ While falls and RTIs continue to be leading causes of injuries, differences in rates of falls versus RTIs may be related to hospital location and proximity to highways. This also may highlight patterns of traumatic injuries trending toward more RTIs due to urbanization.

The study reflects that the younger population are involved in more accidents and trauma accounting for more than 30% of all patients. This is consistent with other studies which also show higher rates of injuries in young adults.^10^ Recognizing the burden in this age group, the implementation of many prevention strategies related to road safety, occupational safety, and violence needs to be considered in Nepal.

Males presented more often with injuries than females. This is similar to findings from other studies done in Nepal, India and other developed countries.^11-13^ This is likely due to males working more often outside the home, and thus travelling via motor vehicles more often, increasing their risk for injury. Additionally, men are more often engaged in labour work, particularly construction which increases the risk of falls and accidents.^1^ Similar results were found in a study in India with most of the patients identifying as labourers (37.7%).^12^

Most injuries seen were related to extremity fractures followed by external injuries, which is similar to other studies done in other developing countries.^13^ Lower extremity fractures are the most common injuries among all the fractures. This may also be due to the increased number of motorcycles used in Nepal over cars, leading to lower extremity injuries.^14^ Additionally, many extremity fractures were related to falls in the elderly population.

The overall mortality rate was comparatively low compared to other studies.^1,9^ This is likely due to the hospital being in an urban area of Nepal, where there are few highways to account for the high-speed mechanism of injury. Additionally, this could be attributed to urban areas having more availability of markets and gas for stoves, resulting in fewer individuals climbing trees for fruits, collecting wood for cooking, or feeding livestock. The majority of critical injuries and deaths in our study were related to head injuries. This is likely due to poor safety helmet usage which is prevalent in developing countries. The use of safety helmets by workers and motorcyclists has been shown to reduce the risk and severity of the injury.^14^

This study was performed in one hospital in an urban setting and therefore may not represent the burden and severity of injury of patients seen in rural areas and along highways. Also, while we attempted to collect data for every trauma patient, without an EMR, we cannot be sure that we captured every patient which might also have caused a low prevalence in the study. We were able to include the most severe cases as all cases that are admitted, referred, or die are recorded in the ED logbooks. This may give us skewed data toward higher rates of admission and referral; however, this allows us to understand the injuries and needs of the most severely injured patients.

## CONCLUSIONS

The prevalence of traumatic injury among patients presenting to emergency was found to be lower than other studies done in similar settings. Trauma registry can be maintained for continuous real time data of trauma patients in tertiary care hospitals.

## References

[1] Pant PR, Banstola A, Bhatta S, Mytton JA, Acharya D, Bhattarai S (2020). Burden of injuries in Nepal, 1990-2017: findings from the Global Burden of Disease Study 2017.. Inj Prev..

[2] Mytton JA, Bhatta S, Thorne M, Pant PR (2019). Understanding the burden of injuries in Nepal: a systematic review of published studies.. Cogent Medicine..

[3] Baker SP, O'Neill B (1976). The injury severity score: an update.. J Trauma..

[4] World Health Organization. (2020). WHO dataset for injury [Internet]..

[5] Gupta S, Wong EG, Nepal S, Shrestha S, Kushner AL, Nwomeh BC (2015). Injury prevalence and causality in developing nations: Results from a countrywide population-based survey in Nepal.. Surgery..

[6] Khanal VK, Upreti R, Oli U, Sunny AK, Ghimire A, Jha N (2017). Prevalence of injury and its associated factors in a rural area of eastern Nepal.. Journal of Chitwan Medical College..

[7] Paudel S, Dhungana S, Pokhrel N, Dhakal GR (2021). Epidemiology of trauma patients presented at emergency department of Trauma Center.. J Nepal Health Res Counc..

[8] Shrestha R, Shrestha SK, Kayastha SR, Parajuli N, Dhoju D, Shrestha D (2013). A comparative study on epidemiology, spectrum and outcome analysis of physical trauma cases presenting to emergency department of Dhulikhel Hospital, Kathmandu University Hospital and its outreach centers in rural area.. Kathmandu Univ Med J (KUMJ)..

[9] Bhatta S, Magnus D, Mytton J, Joshi E, Bhatta S, Adhikari D (2021). The epidemiology of injuries in adults in Nepal: findings from a hospital-based injury surveillance study.. Int J Environ Res Public Health..

[10] Sahdev P, Lacqua MJ, Singh B, Dogra TD (1994). Road traffic fatalities in Delhi: causes, injury patterns, and incidence of preventable deaths.. Accid Anal Prev..

[11] Haagsma JA, Graetz N, Bolliger I, Naghavi M, Higashi H, Mullany EC (2016). The global burden of injury: incidence, mortality, disability-adjusted life years and time trends from the Global Burden of Disease study 2013.. Inj Prev..

[12] Harna B, Arya S, Bahl A (2020). Epidemiology of Trauma Patients Admitted to a Trauma Center in New Delhi, India.. Indian J Crit Care Med..

[13] Aluisio AR, De Wulf A, Louis A, Bloem C (2015). Epidemiology of traumatic injuries in the Northeast Region of Haiti: a cross-sectional Study.. Prehosp Disaster Med..

[14] Liu BC, Ivers R, Norton R, Boufous S, Blows S, Lo SK (2004). Helmets for preventing injury in motorcycle riders.. Cochrane Database Syst Rev..

